# High-performance gallium nitride dielectric metalenses for imaging in the visible

**DOI:** 10.1038/s41598-021-86057-w

**Published:** 2021-03-22

**Authors:** Meng-Hsin Chen, Wei-Ning Chou, Vin-Cent Su, Chieh-Hsiung Kuan, Hoang Yan Lin

**Affiliations:** 1 Graduate Institute of Photonics and Optoelectronics, National Taiwan University, Taipei, 10617 Taiwan; 2 Department of Electrical Engineering, National United University, Miaoli, 36003 Taiwan; 3 Department of Electrical Engineering and Graduate Institute of Electronics Engineering, National Taiwan University, Taipei, 10617 Taiwan

**Keywords:** Materials for devices, Materials for optics, Nanoscale materials, Optics and photonics

## Abstract

Metalens is one of the most promising applications for the development of metasurfaces. A wide variety of materials have been applied to metalenses working at certain spectral bands in order to meet the requirements of high efficiency and low-cost fabrication. Among these materials, wide-bandgap gallium nitride (GaN) is one of the most promising materials considering its advantages especially in semiconductor manufacturing. In this work, GaN has been utilized to fabricate the high-performance metalenses operating at visible wavelengths of 405, 532, and 633 nm with efficiencies up to 79%, 84%, and 89%, respectively. The homemade 1951 United State Air Force (UASF) resolution test chart has also been fabricated in order to provide resolvable lines with widths as small as 870 nm. As shown in the experimental results for imaging, the metalens designed at 405 nm can provide extremely high resolution to clearly resolve the smallest lines with the nano-sized widths in the homemade resolution test chart. These extraordinary experimental results come from our successful development in design and fabrication for the metalenses composed of high-aspect-ratio GaN nanoposts with nearly vertical sidewalls.

## Introduction

Metasurfaces are constructed of artificial patterns with a subwavelength period, making it possible to exhibit exotic electromagnetic properties with high efficiency^1–5^. A wide variety of applications for wavefront shaping have been developed using these novel metasurfaces over the last couple of years^6–21^ and those applications were hard to be realized in conventional optical devices. As one of the most attractive wavefront-engineered applications, ultra-thin metalens^22–24^ has revolutionized the means with which the propagation direction of light can be manipulated by shaping the related wavefront. Several excellent papers focusing on the metalenses made of different materials have been reported, some of which are made of metal structures^25–31^ while others involve only dielectrics^32–40^. However, metalenses made of noble metal structures are known to have strong light attenuation associated with high plasmatic metal loss in the visible. On the other hand, dielectric metalenses are promising to be capable of achieving high transmission efficiency with much less reflection at visible. Various dielectric metalenses have been demonstrated using silicon (Si)^38^, silicon dioxide (SiO_2_)^41^, titanium oxide (TiO_2_)^32^, gallium nitride (GaN)^34^, etc. Among these dielectric materials, GaN prepared by metal organic chemical vapor deposition (MOCVD) should be one of the most promising materials considering its advantages in wide bandgap, low absorption and high refractive index at visible wavelengths, high yield, mass production, and low-cost fabrication.

Recently, we have developed the top-down approach to fabricate three distinct dielectric metalenses composed of GaN dielectric structures with Pancharatnam-Berry phase rotational morphology at visible wavelengths of 430, 532, and 633 nm^34^. Nevertheless, the metalens designed at the wavelength of 633 nm possesses the focusing efficiency as low as 50.6%, mainly due to the imperfection of fabrication processes resulting in conical nanostructures as shown in our previous work^34^. Such tapered nanostructures lead to a large phase error even with a small degree-tapering as demonstrated in the literature^33^, making it hard to realize high efficiency and diffraction-limited focusing for the metalens. Moreover, the lack of evaluation of the imaging performance for these metalenses limits their applications at visible wavelengths in future markets.

In this study, we demonstrate high-performance metalenses designed and fabricated for working at visible wavelengths of 405, 532, and 633 nm. We employ the top-down approach to accomplish high-aspect-ratio dielectric GaN nanoposts with nearly vertical sidewalls in order to build up these metalenses capable of focusing incident visible light beams into diffraction-limited spots. The efficiency for the metalens designed at the wavelength of 633 nm has been experimentally achieved as high as 89%, which is close to the simulated efficiency of 90.3%. To evaluate the practical imaging performance of our metalenses, the 1951 United State Air Force (UASF) resolution test chart was utilized as our test target. With the 405-nm-desinged metalens, the smallest features in the test chart that we can observe are lines with widths as small as 870 nm, which is the best resolution ever achieved for the metalens with the numerical aperture (NA) of 0.3 as far as we know, and never attempted before this work.

## Metalens design

**Figure 1:**
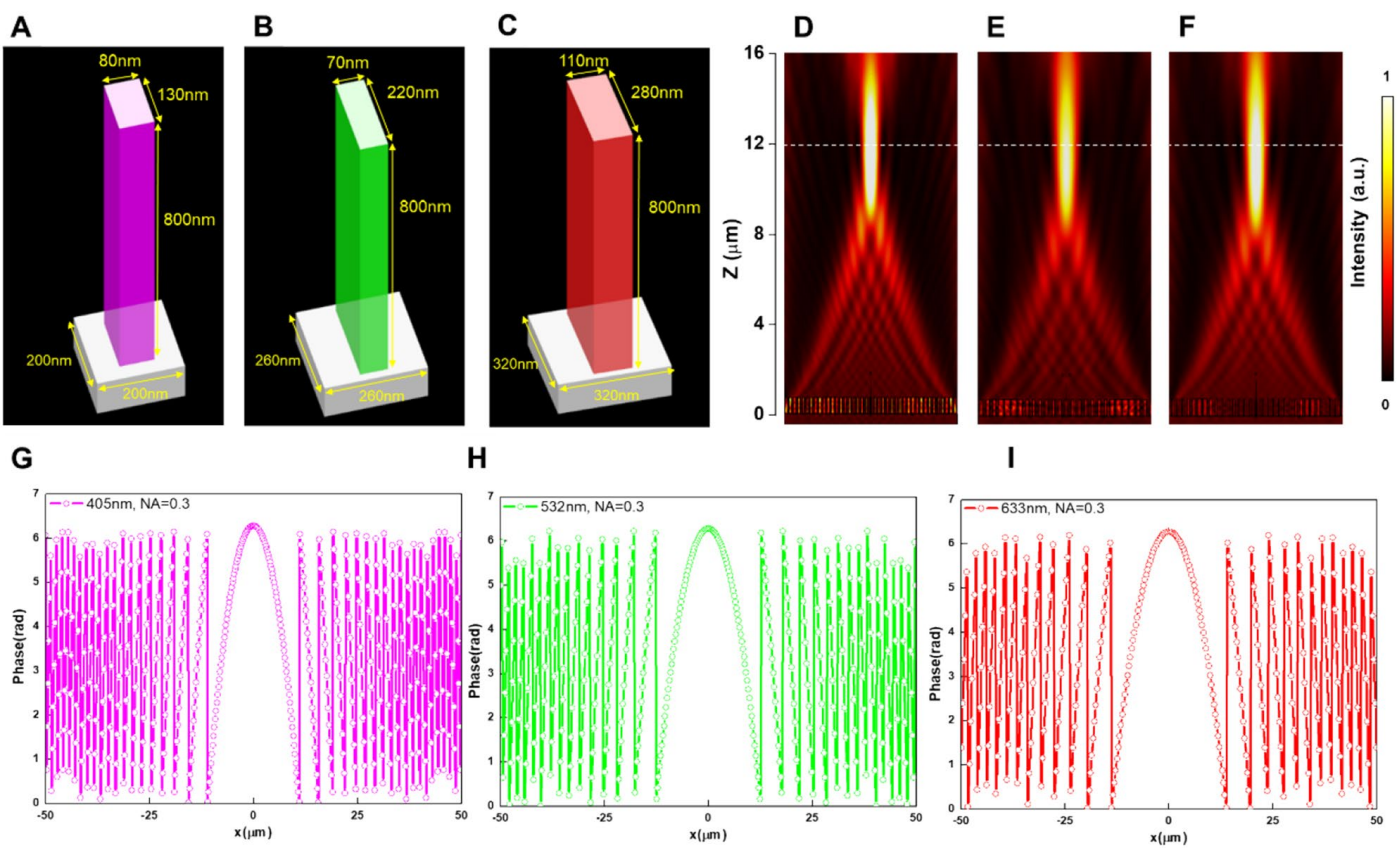
Design and simulation of metalenses. (**A**–**C**) Schematics of the metalenses building blocks designed at wavelengths of (**A**) 405, (**B**) 532, and (**C**) 633 nm. (**D**–**F**) Numerical intensity profiles of the metalenses designed at wavelengths of (**D**) 405, (**E**) 532, and (F) 633 nm. (**G**–**I**) Phase profiles for the metalenses designed at wavelengths of (**G**) 405, (**H**) 532, and (**I**) 633 nm.

Figure 1A–C, shows schematic figures of the building blocks with respect to the given subwavelength periods of 200, 260, and 320 nm for metalenses operating at three distinct wavelengths of 405, 532, and 633 nm, respectively. The structural parameters of each building block have been numerically optimized at the design wavelength to achieve the highest operating efficiency corresponding to the respective subwavelength period as shown in Fig. S1 in Supplementary Materials. The phase retardation distribution of a converging lens can be described as:

1 $$\mathrm{\varphi }\left(\mathrm{x},\mathrm{y}\right)=\left(f-\sqrt{{d}^{2}+{f}^{2}}\right)\frac{2\pi }{\lambda },$$

where $f$ and $\lambda$ are respectively the design focal length and the operating wavelength in free space. $d=\sqrt{{x}^{2}+{y}^{2}}$ is the distance between the location of each nanopost and the center of the metalens surface. Such phase profile can be readily implemented by introducing the simple design principle of the Pancharatnam-Berry phase method^32,34^ through rotating the orientation of nanoposts composed of identical feature sizes to reach each phase shift at the design location. The simulated cross-sectional intensity distributions for the metalenses designed at wavelengths of 405, 532, and 633 nm with a design focal length of 12 μm and the NA of 0.3 are shown in Fig. 1D–F, revealing that the focal lengths remain at the design position, which are consistent with our design. Smaller diameter and focal distance used for metalenses simulation here can be ascribed to the limited memory and computation ability of our computer. Moreover, the simulated conversion efficiencies calculated as the ratio of the optical power of focused light divided by optical power of total incident light have achieved to be as high as 81.1%, 86.4%, and 90.3% for metalenses at the designed wavelengths of 405, 532, and 633 nm, respectively.

## Fabrication and measurement results

To demonstrate device performance for practical focusing and imaging, we fabricated three distinct metalenses that possess optimally structural dimensions as simulated above. Details for fabrication parameters and processes can be found in the Methods section. All of these fabricated metalenses impart the same diameter of 100 μm and focal length of 150 μm, yielding the same NA of 0.3 as our aforementioned simulation results in order to compare the device performance. Figure 1G–I, point out the required phase profiles for the three distinct metalenses, showing that more nanostructures are needed to construct the metalens designed at a shorter wavelength.

**Figure 2:**
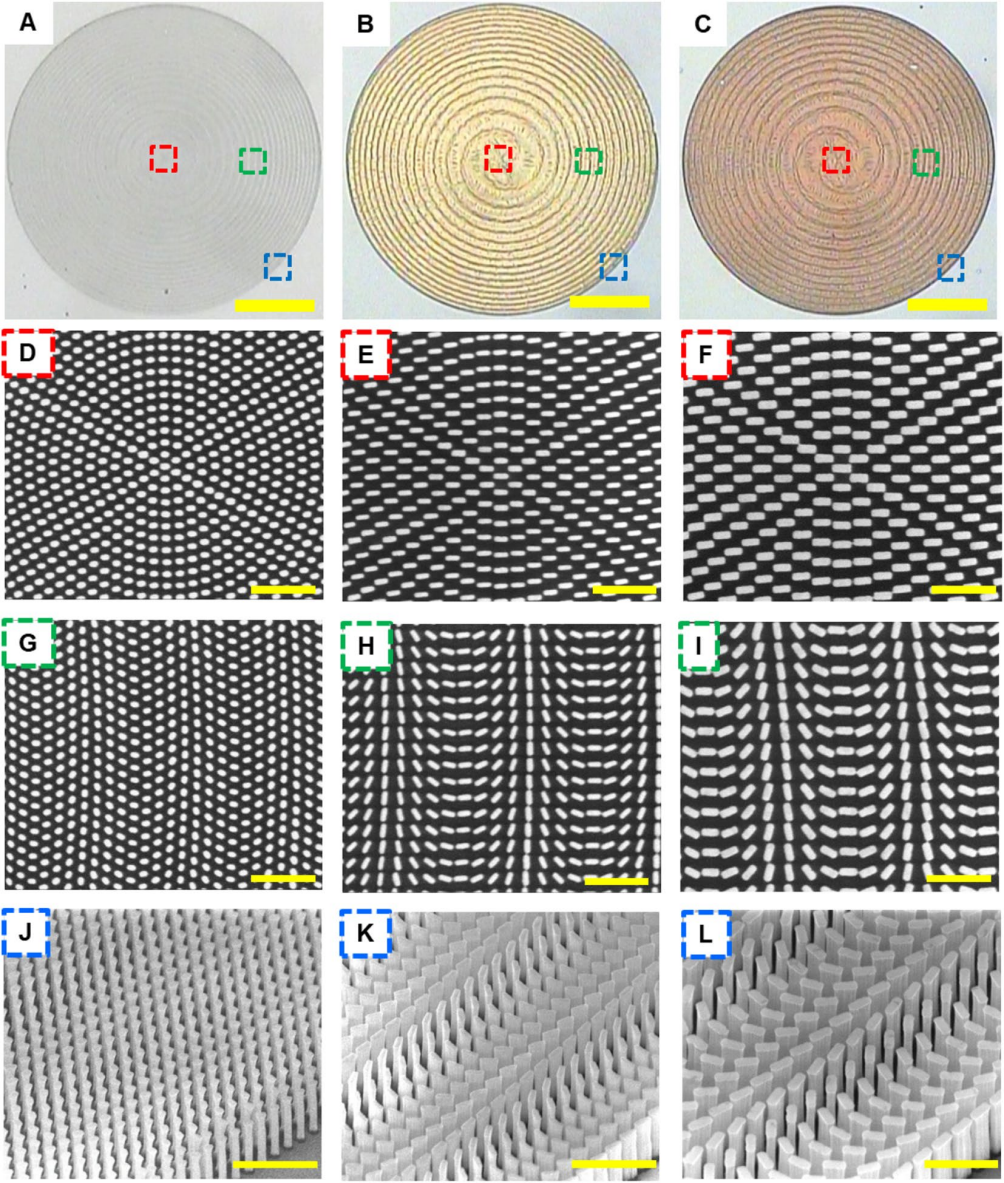
Micrographs of the metalenses. (**A**–**C**) Optical images of the fabricated metalenses designed at wavelengths of (**A**) 405, (**B**) 532, and (**C**) 633 nm. Scale bar: 25 μm. (**D**–**I**) The top-view SEM images shown in (**D**,**G**), (**E**,**H**), and (**F**,**I**) corresponding to the highlighted regions in (**A**–**C**), respectively. (**J**–**L**) The tilt-view SEM images shown in (**J**), (**K**), and (**L**) corresponding to the highlighted regions in (**A**–**C**). Scale bar, 1 μm in (**D**–**L**).

Figure 2A–C, show the top-view of optical microscope (OM) images of fabricated metalenses working at the respective wavelengths. And the top-view of zoom-in scanning electron microscope (SEM) images of corresponding metalens samples are illustrated in Fig. 2D–I. From inspections of these OM and SEM images, all of nanoposts in these metalenses have been well-defined after several pattern transfer techniques, showing accurate fabrication processes for nano-sized structures. Figure 2J–L, present the tilt-view of zoom-in SEM images for associated metalenses, where nanoposts in each figure preserve high aspect ratio with nearly vertical sidewalls. It is worth noticing that the degree of complexity in fabricating such nano-sized structures made of GaN is very challenging to be realized in practice by using the top-down fabrication approach considering inevitably lateral dimension etching.

**Figure 3:**
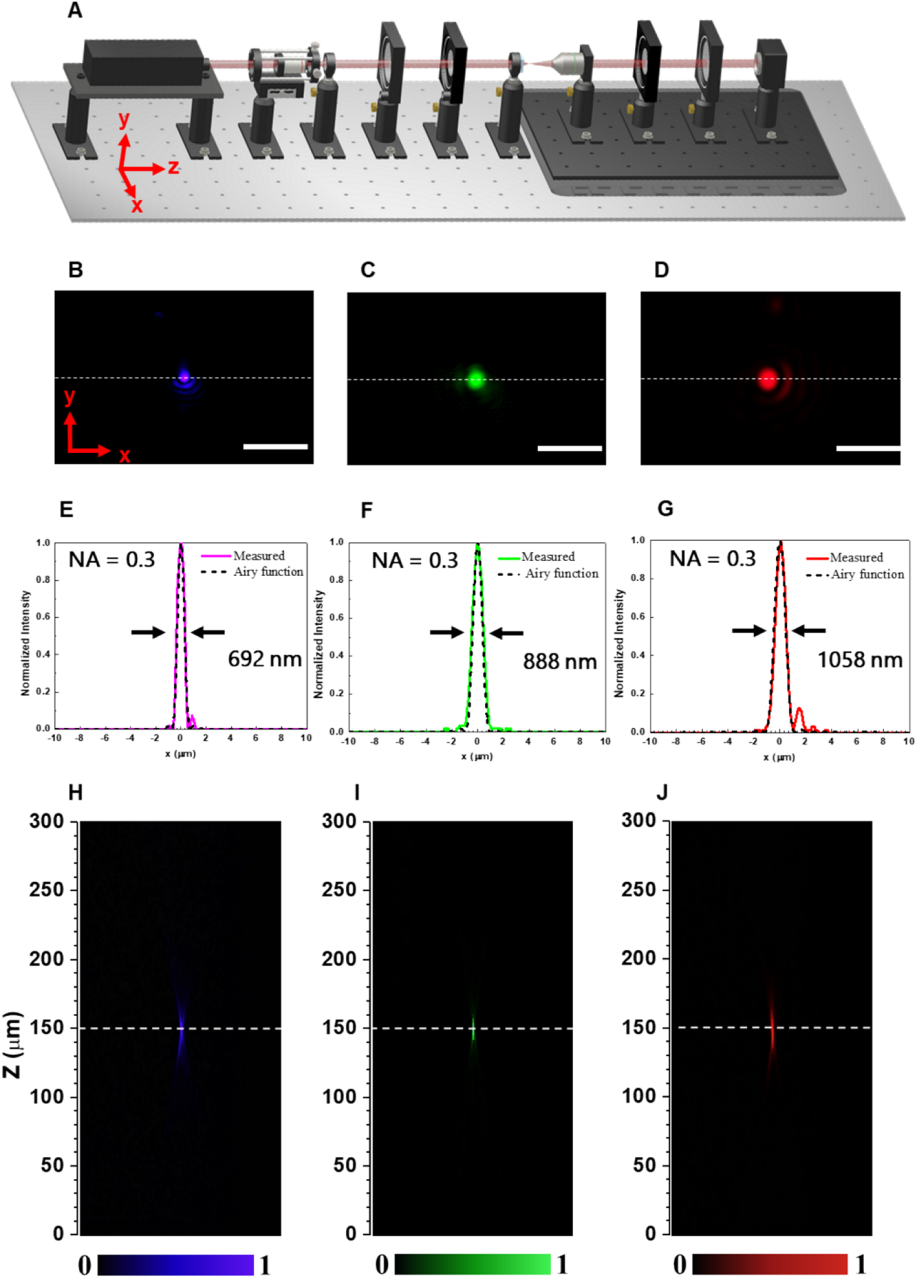
Characterization of the metalenses. (**A**) Measurement setup for metalenses characterization. (**B**–**D**) Measured focal spots of the metalenses designed at wavelengths of (**B**) 405, (**C**) 532, and (**D**) 633 nm. Scale bar: 5 μm. (**E**–**G**) Corresponding horizontal cuts of focal spots with FWHM of 692, 888, and 1058 nm. Dashed lines referring to normalized ideal Airy function. (**H**–**J**) Intensity profiles of the metalenses along axial planes designed at wavelengths of (**H**) 405, (**I**) 532, and (**J**) 633 nm.

The experimental setup for measuring focal spot profiles and efficiencies of the metalenses are illustrated in Fig. 3A. Here, we use a laser beam to sequentially go through a spatial filter, a polarizer, and a quarter-wave plate in order to produce circularly polarized light of high quality. An objective lens is used to image the light focused by a metalens, which is equipped with an electric motional stage to adjust the distance between the objective lens and a metalens. Then a quarter-wave plate and a polarizer are used to convert circularly polarized light into linearly polarized light. Finally, a complementary metal oxide semiconductor (CMOS) camera is used to record an image of the focused light.

Figure 3B–G, demonstrate the experimental results of focal spot profiles and associated cross-sectional distributions in comparison with the normalized Airy function (dashed lines) for the three distinct metalenses, respectively. All of the metalenses show highly symmetric focusing profiles with the circularly-polarized incident visible light beams. The cross-sectional distributions for all metalenses are extremely close to theoretically normalized Airy function, displaying tremendous performance in light convergence for all metalenses. To evaluate the diffraction-limited focusing abilities, the corresponding full-widths at half-maximum (FWHM) of focal spot profiles for the metalenses designed at distinct wavelengths of 405, 532, and 633 nm have been measured as 692, 888, 1058 nm, respectively, which are close to theoretical diffraction-limited values $\left(\frac{\lambda }{2NA}\right).$ The results demonstrate good converging functions as being focusing lenses free of spherical aberration. The measured efficiencies defined as the ratio of focused optical power with opposite helicity to the total incident optical power with circularly polarized incident light for these metalenses are 79% (405 nm), 84% (532 nm), and 89% (633 nm). It is worth mentioning that the achievable focusing efficiency for 633-nm-designed metalens composed of the tapered nanostructures is as low as 50.6% as shown in our previous work^34^. The total incident optical power was measured by a power meter via light passing through a pin hole with the same diameter of 100 μm as designed in the fabricated metalenses. These measured efficiencies for the fabricated metalenses are also in good agreement with simulated efficiencies as demonstrated above, showing a successful development in precise fabrication processes. Moreover, the cross-sectional light intensity profiles along the propagation direction of incidences for theses metalenses haven been performed in Fig. 3H–J, providing another evidence of successful metalens fabrication that the focal lengths measured for all of the metalenses remain in the same position as the design focal length. The setup for this optical measurement has the same configuration as illustrated in Fig. 3A. One can see Fig. S3 in Supplementary Materials for the experiments of the depth of focus (DOF) measured with the metalenses working for visible light. The experimental results point out that an increase in the measured values of DOFs with the use of the metalenses designed at longer wavelengths.

**Figure 4:**
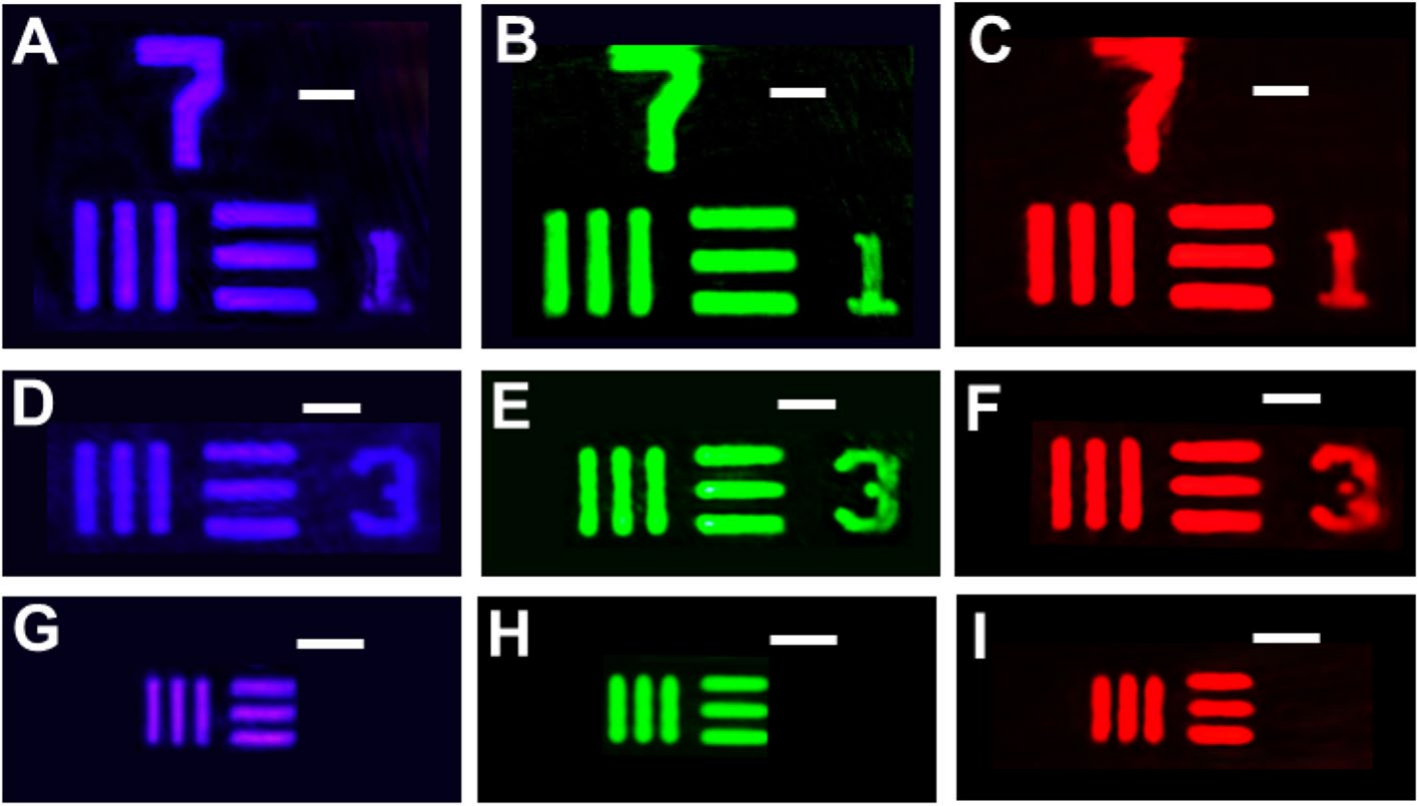
Imaging with the metalenses. (**A**–**I**) Images of the 1951 USAF resolution test chart formed by the metalenses at laser wavelengths of (**A**,**D**,**G**) 405, (**B**,**E**,**H**) 532, and (**C**,**F**,**I**) 633 nm. (**A**–**C**) The smallest features are lines with widths of 3.9 µm and center-to-center distances of 7.8 µm. (**D**–**F**) The smallest features are lines with widths of 3.1 µm and center-to-center distances of 6.2 µm. (**G**–**I**) The smallest features are lines with widths of 2.2 µm and center-to-center distances of 4.4 µm. Scale bar, 10 μm in (**A**–**I**).

To perform practical capabilities of imaging for these metalenses, we first choose a commercially produced device purchased from Thorlab Inc., the 1951 USAF resolution test chart, as our target for imaging. Such purchased resolution test chart is composed of elements consisting of three vertical and horizontal bars with the same length, width, and space, providing the smallest lines with widths of 2.2 μm. The measurement system is revealed in the Methods section. Images taken from a CMOS camera and formed by the three metalenses operating at wavelengths of 405, 532, and 633 nm, respectively, can be demonstrated in Fig. 4A–I. All of the images taken from the three metalenses show remarkably clear and sharp line features, even the smallest features in the present purchased USAF resolution test chart can be clearly resolved as well. It is worth mentioning that the resolution of the metalenses is capable of distinguishing micro-sized features clearly at visible wavelengths, which can be ascribed to the successful fabrication of these well-functioned metalenses composed of the nanoposts with nearly vertical sidewalls.

**Figure 5:**
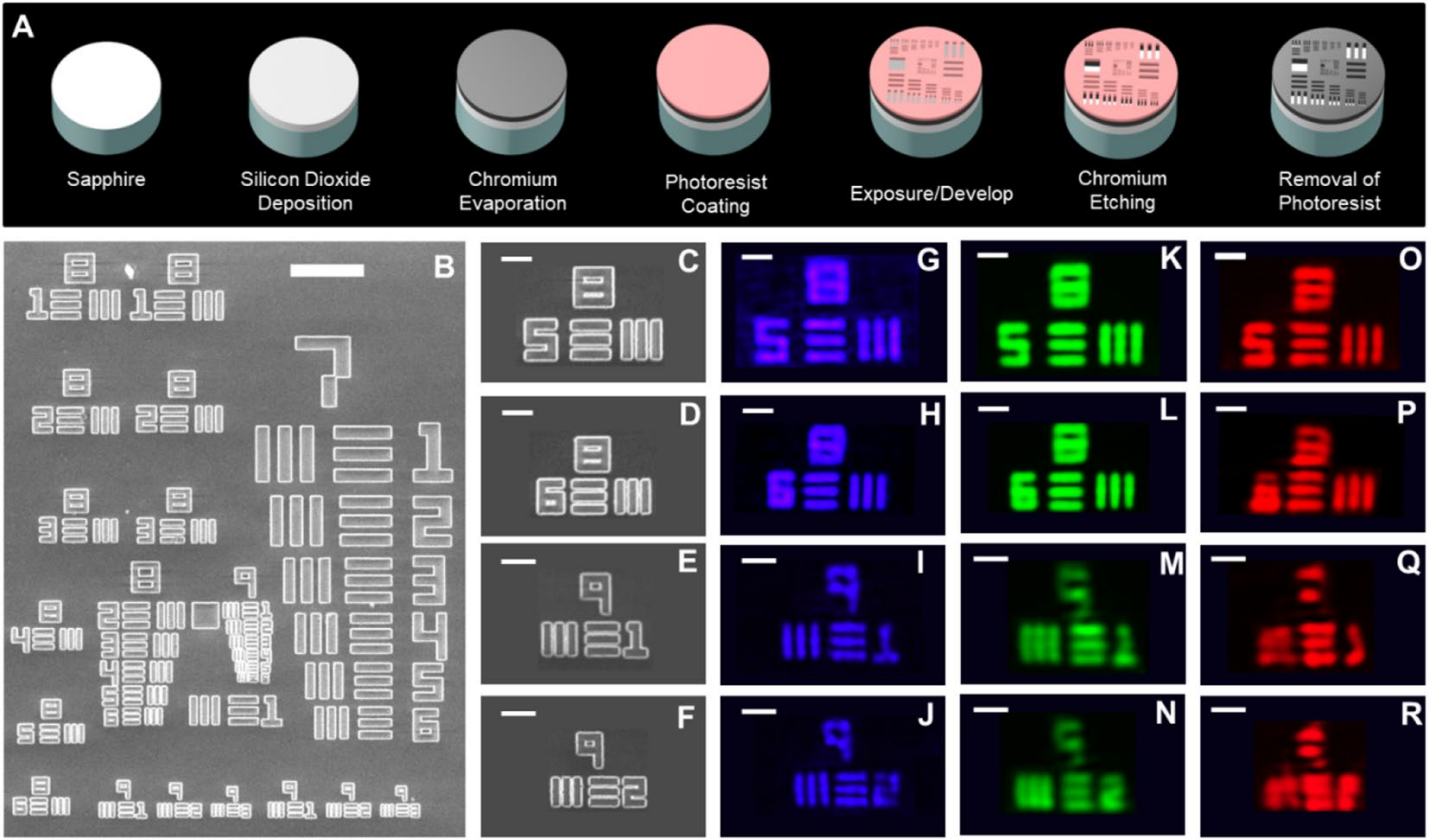
Imaging with the metalens. (**A**) Fabrication of the 1951 USAF resolution test chart. (**B**) SEM micrograph of the fabricated 1951 USAF resolution test chart. Scale bar: 25 μm. (**C**–**F**) SEM micrographs of the USAF element. (**C**) Group 8 element 5, (**D**) Group 8 element 6, (**E**) Group 9 element 1, and (**F**) Group 9 element 2. (**G**–**R**) Corresponding Images of the USAF elements formed by the metalenses at laser wavelengths of (**G**–**J**) 405, (**K**–**N**) 532, and (**O**–**R**) 633 nm. Scale bar, 5 μm in (**C**–**R**).

To demonstrate the capability of diffraction-limited imaging for our metalenses, we have prepared our homemade 1951 USAF resolution test chart that offers resolvable lines with widths as small as 870 nm. Figure 5A depicts the schematic figures of our fabrication processes. The processes start with a double-polished sapphire substrate. A SiO_2_ layer is then deposited on the substrate. After that, a 350-nm-thick chromium (Cr) layer is evaporated on the SiO_2_-deposited sapphire substrate followed by spin-coating a resist layer directly onto the top surface of the substrate. The resist-coated substrate is exposed to concentrated electron beam and a wet etching process is performed to build up lines of our homemade test chart after the development process. The sample fabrication will be accomplished after the removal of a resist layer.

Dimensions of each line in our homemade test chart have been carefully examined by use of the focused-ion beam system with high resolution imaging capability in order to meet requirements for industrial standards for accuracy. Figure 5B, shows the top-view of the SEM image of the fabricated 1951 USAF resolution test chart. And the zoom-in SEM images proving the fabricated lines with correct feature sizes are presented in Fig. 5C–F. The measurement configuration is similar to the one shown in the Methods section, except that the imaging target has been replaced by the homemade one. Figure 5G–R, demonstrate the imaging results for the fabricated metalenses. One can expect that the images taken with the metalenses working at the longest wavelength of 633 nm firstly become blur when we compare with other metalenses. No surprisingly, the contrast significantly drops for the metalens designed at 633 nm when we begin imaging lines with widths smaller than 1.1 μm (Fig. 5P) because of the diffraction limit. The images taken with the green one maintain resolvable until the widths of the lines reach 0.98 μm as shown in Fig. 5M. Nevertheless, the edges of lines with widths in Fig. 5G–J, still remain exceptionally clear line features as small as 870 nm for the metalens working at the shortest wavelength of 405 nm with the NA of 0.3. The results agree with the metalens working at a shorter wavelength shows superior resolution capability under the premise that complex fabrication processes have been successfully developed.

## Conclusion

In this work, we have demonstrated high-performance metalenses comprised of GaN nanoposts. Using Pancharanam-Berry phase rotational morphology, the metalenses are capable of forming diffraction-limited focal spots at design wavelengths and achieving experimentally efficiency as high as 89%. The imaging capabilities of these metalenses can provide extremely high resolution as demonstrated by resolving the smallest lines with the nano-sized widths in the homemade 1951 USAF resolution test chart. These tremendous devices performance can be attributed to successful development in designing and fabricating the metalenses composed of the high-aspect-ratio GaN nanoposts with nearly vertical sidewalls. This work can provide opportunities for the semiconductor industry to participate in developing visible optical components using dielectric metasurfaces based on the wide-bandgap GaN material.

## Methods

### Details of fabrication parameters and processes

A 800-nm-thick gallium nitride (GaN) layer is grown on a sapphire substrate by metal organic chemical vapor deposition (MOCVD). A 400-nm-thick silicon dioxide (SiO_2_) layer is then deposited by plasma-enhanced chemical vapor deposition (PECVD). After that, a 100-nm-thick resist (ZEP520A) layer is spin coated on the SiO_2_ layer, and electron beam lithography is utilized to create the pattern in the resist layer with the exposure and develop processes. A 40-nm-thick chromium (Cr) layer is deposited on the substrate by electron-beam evaporation, and a lift-off process is implemented afterwards. Then, the SiO_2_ is etched by reactive-ion etching (RIE) using the Cr layer as a hard mask, followed by the removal of the Cr layer with the CR-7 chromium etchant. The GaN layer is subsequently etched by inductively coupled plasma reactive-ion etching (ICP-RIE) with the patterned SiO_2_ layer as a hard mask. The sample fabrication will be accomplished after the SiO_2_ layer is removed with the buffered oxide etch (BOE) solution. The schematics for the metalens fabrication processes can be found in Supplementary Materials, Fig. S2.

### The 1951 United State Air Force (USAF) resolution test chart measurements

The laser beam first passes through a spatial filter and a plano-convex lens to form higher-quality laser light. The laser light passes through a polarizer and a quarter-wave plate to produce circularly polarized light afterwards. A 10× objective lens is successively used to focus the light onto the USAF 1951 target. A 20× objective lens is used to image the light focused by a metalens. The distance between the 20× objective lens and the metalens can be adjusted by using an electric stage. Then a quarter-wave plate and a polarizer are used to convert circularly polarized light into linearly polarized light. Finally, a CMOS camera is used to record an image of the 1951 USAF resolution test chart. The schematics of the present measurement setup can be shown in Supplementary Materials, Fig. S4.

## Supplementary information

Supplementary information.

## Data Availability

The data regarding the findings of this study are available from the corresponding authors.

## References

[1] Zheludev NI, Kivshar YS (2012). From metamaterials to metadevices. Nat. Mater..

[2] Kildishev AV, Boltasseva A, Shalaev VM (2013). Planar photonics with metasurfaces. Science.

[3] Meinzer N, Barnes WL, Hooper IR (2014). Plasmonic meta-atoms and metasurfaces. Nat. Photonics.

[4] Yu N, Capasso F (2014). Flat optics with designer metasurfaces. Nat. Mater..

[5] Su VC, Chu CH, Sun G, Tsai DP (2018). Advances in optical metasurfaces: Fabrication and applications invited. Opt. Express.

[6] Sun S, He Q, Xiao S, Xu Q, Li X, Zhou L (2012). Gradient-index meta-surfaces as a bridge linking propagating waves and surface waves. Nat. Mater..

[7] Aieta F, Kats MA, Genevet P, Capasso F (2015). Multiwavelength achromatic metasurfaces by dispersive phase compensation. Science.

[8] Khorasaninejad M, Aieta F, Kanhaiya P, Kats MA, Genevet P, Rousso D, Capasso F (2015). Achromatic metasurface lens at telecommunication wavelengths. Nano Lett..

[9] Ding X, Monticone F, Zhang K, Zhang L, Gao D, Burokur SN, de Lustrac A, Wu Q, Qiu CW, Alu A (2015). Ultrathin pancharatnam-berry metasurface with maximal cross-polarization efficiency. Adv. Mater..

[10] Arbabi A, Horie Y, Bagheri M, Faraon A (2015). Dielectric metasurfaces for complete control of phase and polarization with subwavelength spatial resolution and high transmission. Nat. Nanotechnol..

[11] High AA, Devlin RC, Dibos A, Polking M, Wild DS, Perczel J, de Leon NP, Lukin MD, Park H (2015). Visible-frequency hyperbolic metasurface. Nature.

[12] Wang B, Dong F, Li QT, Yang D, Sun C, Chen J, Song Z, Xu L, Chu W, Xiao YF, Gong Q, Li Y (2016). Visible-frequency dielectric metasurfaces for multiwavelength achromatic and highly dispersive holograms. Nano Lett..

[13] Khorasaninejad M, Chen WT, Zhu AY, Oh J, Devlin RC, Rousso D, Capasso F (2016). Multispectral chiral imaging with a metalens. Nano Lett..

[14] Arbabi E, Arbabi A, Kamali SM, Horie Y, Faraon A (2017). Controlling the sign of chromatic dispersion in diffractive optics with dielectric metasurfaces. Optica.

[15] Wei QS, Sain B, Wang YT, Reineke B, Li XW, Huang LL, Zentgraf T (2019). Simultaneous spectral and spatial modulation for color printing and holography using all-dielectric metasurfaces. Nano Lett..

[16] Wang Z, Li T, Soman A, Mao D, Kananen T, Gu T (2019). On-chip wavefront shaping with dielectric metasurface. Nat. Commun..

[17] Yang W, Xiao S, Song Q, Liu Y, Wu Y, Wang S, Yu J, Han J, Tsai DP (2020). All-dielectric metasurface for high-performance structural color. Nat. Commun..

[18] Ni Y, Chen S, Wang Y, Tan Q, Xiao S, Yang Y (2020). Metasurface for structured light projection over 120 degrees field of view. Nano Lett..

[19] Yuan YY, Sun S, Chen Y, Zhang K, Ding XM, Ratni B, Wu Q, Burokur SN, Qiu CW (2020). A fully phase-modulated metasurface as an energy-controllable circular polarization router. Adv. Sci..

[20] Yuan YY, Zhang K, Ratni B, Song QH, Ding XM, Wu Q, Burokur SN, Genevet P (2020). Independent phase modulation for quadruplex polarization channels enabled by chirality-assisted geometric-phase metasurfaces. Nat. Commun..

[21] Zhang K, Yuan YY, Ding XM, Li HY, Ratni B, Wu Q, Liu J, Burokur SN, Tan JB (2021). Polarization-engineered noninterleaved metasurface for integer and fractional orbital angular momentum multiplexing. Laser Photon. Rev..

[22] Lin RJ, Su VC, Wang SM, Chen MK, Chung TL, Chen YH, Kuo HY, Chen JW, Chen J, Huang YT, Wang JH, Chu CH, Wu PC, Li T, Wang ZL, Zhu SN, Tsai DP (2019). Achromatic metalens array for full-colour light-field imaging. Nat. Nanotechnol..

[23] Engelberg J, Levy U (2020). The advantages of metalenses over diffractive lenses. Nat. Commun..

[24] Li L, Liu ZX, Ren XF, Wang SM, Su VC, Chen MK, Chu CH, Kuo HY, Liu BH, Zang WB, Guo GC, Zhang LJ, Wang ZL, Zhu SN, Tsai DP (2020). Metalens-array-based high-dimensional and multiphoton quantum source. Science.

[25] Yu NF, Genevet P, Kats MA, Aieta F, Tetienne JP, Capasso F, Gaburro Z (2011). Light propagation with phase discontinuities: Generalized laws of reflection and refraction. Science.

[26] Blanchard R, Aoust G, Genevet P, Yu NF, Kats MA, Gaburro Z, Capasso F (2012). Modeling nanoscale V-shaped antennas for the design of optical phased arrays. Phys. Rev. B.

[27] Ni XJ, Emani NK, Kildishev AV, Boltasseva A, Shalaev VM (2012). Broadband light bending with plasmonic nanoantennas. Science.

[28] Sun S, Yang KY, Wang CM, Juan TK, Chen WT, Liao CY, He Q, Xiao S, Kung WT, Guo GY, Zhou L, Tsai DP (2012). High-efficiency broadband anomalous reflection by gradient meta-surfaces. Nano Lett..

[29] Chen X, Huang L, Muhlenbernd H, Li G, Bai B, Tan Q, Jin G, Qiu CW, Zhang S, Zentgraf T (2012). Dual-polarity plasmonic metalens for visible light. Nat. Commun..

[30] Pors A, Nielsen MG, Eriksen RL, Bozhevolnyi SI (2013). Broadband focusing flat mirrors based on plasmonic gradient metasurfaces. Nano Lett..

[31] Wang SM, Wu PC, Su VC, Lai YC, Chu CH, Chen JW, Lu SH, Chen J, Xu BB, Kuan CH, Li T, Zhu SN, Tsai DP (2017). Broadband achromatic optical metasurface devices. Nat. Commun..

[32] Khorasaninejad M, Chen WT, Devlin RC, Oh J, Zhu AY, Capasso F (2016). Metalenses at visible wavelengths: Diffraction-limited focusing and subwavelength resolution imaging. Science.

[33] Khorasaninejad M, Zhu AY, Roques-Carmes C, Chen WT, Oh J, Mishra I, Devlin RC, Capasso F (2016). Polarization-insensitive metalenses at visible wavelengths. Nano Lett..

[34] Chen BH, Wu PC, Su VC, Lai YC, Chu CH, Lee IC, Chen JW, Chen YH, Lan YC, Kuan CH, Tsai DP (2017). GaN metalens for pixel-level full-color routing at visible light. Nano Lett..

[35] Khorasaninejad M, Shi Z, Zhu AY, Chen WT, Sanjeev V, Zaidi A, Capasso F (2017). Achromatic metalens over 60 nm bandwidth in the visible and metalens with reverse chromatic dispersion. Nano Lett.

[36] Paniagua-Dominguez R, Yu YF, Khaidarov E, Choi S, Leong V, Bakker RM, Liang X, Fu YH, Valuckas V, Krivitsky LA, Kuznetsov AI (2018). A metalens with a near-unity numerical aperture. Nano Lett..

[37] Wang SM, Wu PC, Su VC, Lai YC, Chen MK, Kuo HY, Chen BH, Chen YH, Huang TT, Wang JH, Lin RM, Kuan CH, Li T, Wang ZL, Zhu SN, Tsai DP (2018). A broadband achromatic metalens in the visible. Nat. Nanotechnol..

[38] Liang HW, Lin QL, Xie XS, Sun Q, Wang Y, Zhou LD, Liu L, Yu XY, Zhou JY, Krauss TF, Li JT (2018). Ultrahigh numerical aperture metalens at visible wavelengths. Nano Lett..

[39] Yoon G, Kim K, Huh D, Lee H, Rho J (2020). Single-step manufacturing of hierarchical dielectric metalens in the visible. Nat. Commun..

[40] Ndao A, Hsu L, Ha J, Park JH, Chang-Hasnain C, Kante B (2020). Octave bandwidth photonic fishnet-achromatic-metalens. Nat. Commun..

[41] Park JS, Zhang SY, She A, Chen WT, Lin P, Yousef KMA, Cheng JX, Capasso F (2019). All-glass, large metalens at visible wavelength using deep-ultraviolet projection lithography. Nano Lett..

