# Supplementary material for: High-performance gallium nitride dielectric metalenses for imaging in the visible
Source: Sci Rep. 2021 Mar 22;11:6500. doi: 10.1038/s41598-021-86057-w (PMC7985212; doi:10.1038/s41598-021-86057-w)
Supplement: Supplementary file 1 — Supplementary information. [file 41598_2021_86057_MOESM1_ESM.pdf]

Supplementary Materials for

High-performance gallium nitride dielectric metalenses for imaging in the visible

Meng-Hsin Chen[1], Wei-Ning Chou[2], Vin-Cent Su[2†], Chieh-Hsiung Kuan[1,3], and Hoang Yan Lin[1†]

[1]*Graduate Institute of Photonics and Optoelectronics, National Taiwan University, Taipei 10617, Taiwan.*

[2]*Department of Electrical Engineering, National United University, Miaoli 36003, Taiwan*

[3]*Department of Electrical Engineering and Graduate Institute of Electronics Engineering, National Taiwan University, Taipei 10617, Taiwan*

†Corresponding authors: hoangyanlin@ntu.edu.tw; chkuan@ntu.edu.tw; vcsu@nuu.edu.tw

Device simulation

Details of simulation parameters

The CST Microwave Studio was used to carry out all of the simulation results in this study. Figure S1 shows the conversion efficiency of the building blocks optimized for the metalenses operating at wavelengths of 405, 532, 633nm in the visible, respectively. The structural parameters of each building block have been optimized so as to achieve the highest operating efficiency at the design wavelength.

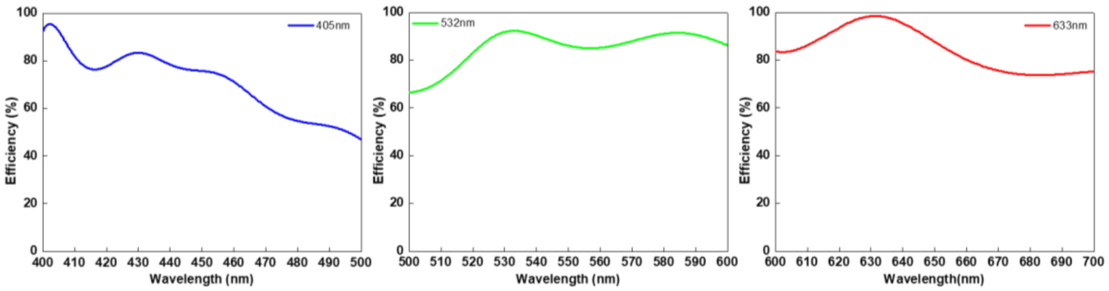

Fig. S1: Simulation efficiencies of the metalenses designed at wavelengths of 405, 532, and 633 nm.

Sample fabrication

We use the electron beam lithography system along with the dry-etching processes to fabricate the metalenses. The schematic figures for the fabrication processes of the metalenses are shown as follows.

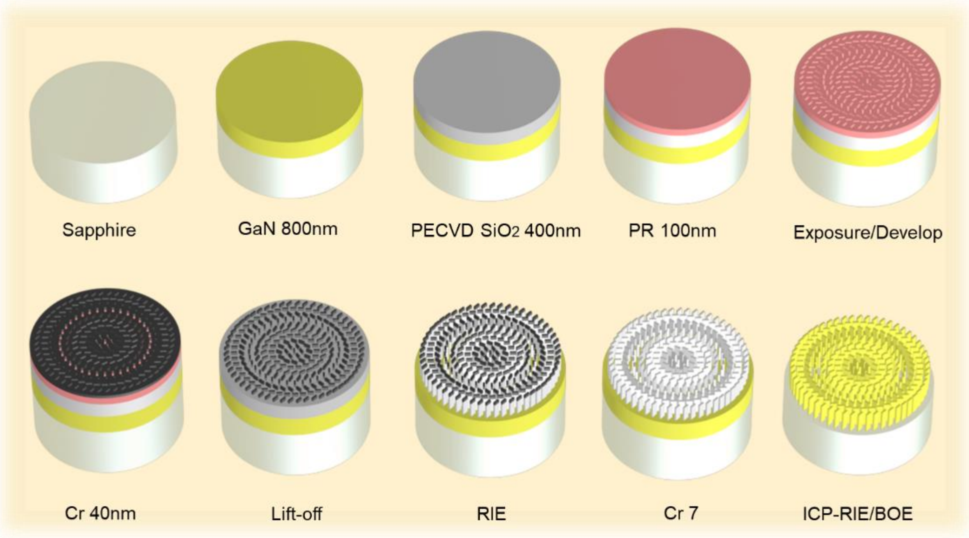

Fig. S2: The process flow for fabricating metalenses.

Experiments

Depth of focus (DOF) measurements

The experimental setup for DOF measurements is the same as the setup for measuring focal spot profiles of the metalenses illustrated in Fig. 3A. An objective lens is used to image the light focused by a metalens. The distance between the objective lens and a metalens can be adjusted by using an electric stage. We first locate the focal spot of the metalens and then measure the intensity profiles of the focal plane along axial planes by successively move away from the focal plane. Finally, a complementary metal oxide semiconductor (CMOS) camera is used to record images within a 30-μm span around the focal point. Fig. S3 illustrated the focal plane intensity profiles along axial planes of the metalens designed at wavelengths of 405, 532, and 633 nm. Experimental results show that the DOF increases as the wavelength increases, which is consistent with the theory.

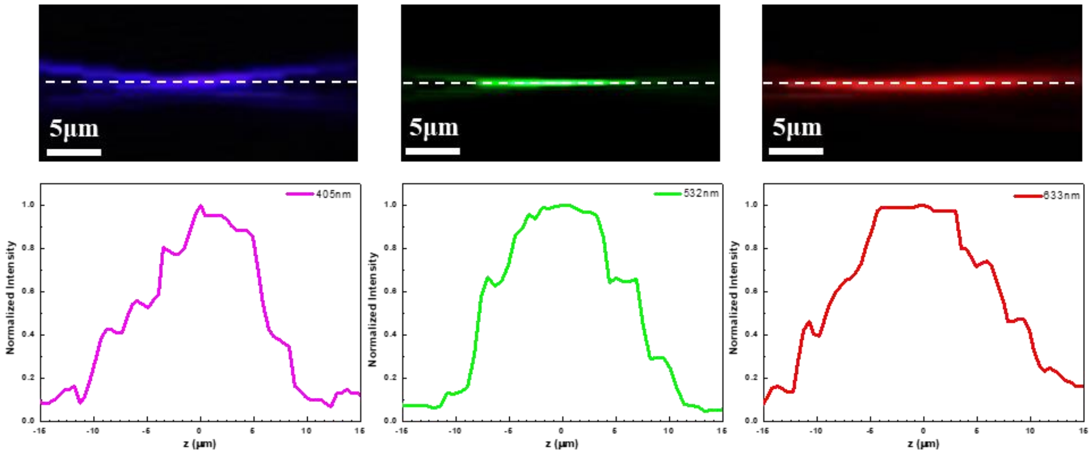

Fig. S3: Intensity profiles along axial planes of the metalens designed at wavelengths of 405, 532, and 633 nm.

The 1951 United State Air Force (USAF) resolution test chart measurements

The schematic for the home-made 1951 USAF imaging is illustrated here.

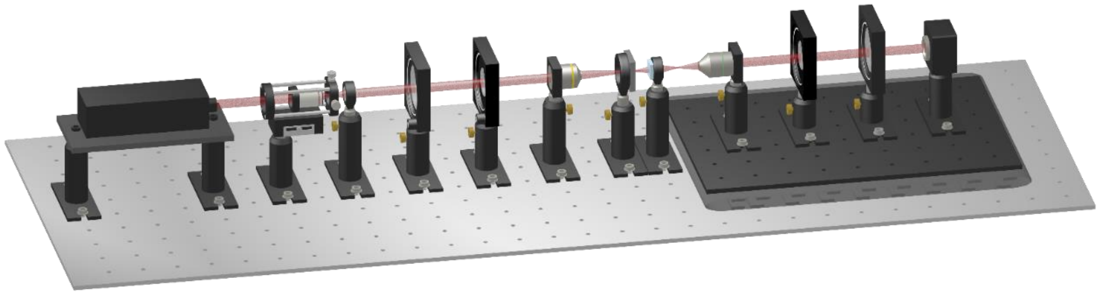

Fig. S4: The experimental setup for imaging.
